# Hierarchical Metal–[Carbon Nitride *Shell*/Carbon *Core*] Electrocatalysts: A Promising New General Approach to Tackle the ORR Bottleneck in Low-Temperature Fuel Cells

**DOI:** 10.1021/acscatal.2c03723

**Published:** 2022-09-27

**Authors:** Vito Di Noto, Enrico Negro, Bhushan Patil, Francesca Lorandi, Soufiane Boudjelida, Yannick H. Bang, Keti Vezzù, Gioele Pagot, Laura Crociani, Angeloclaudio Nale

**Affiliations:** † Section of Chemistry for the Technology (ChemTech), Department of Industrial Engineering, University of Padova, Via Marzolo 9, I-35131 Padova, Italy; ‡ National Interuniversity Consortium of Materials Science and Technology (INSTM), Via Giusti, 9, I-50121 Firenze, Italy; § Consiglio Nazionale delle Ricerche, Istituto di Chimica della Materia Condensata e di Tecnologie per l’Energia, Corso Stati Uniti 4, I-35127 Padova, Italy

## 1 Introduction

The practical implementation of the *hydrogen economy* plays a critical role in the energy transition scenario.^1^ The two pillars of a *green* hydrogen economy are fuel cells (FCs) and electrolyzers (ELs). Low-temperature FCs fed with hydrogen are a key technology to achieve a decarbonized society. In particular, the technology of proton-exchange membrane fuel cells (PEMFCs) is very suitable and almost mature for a large-scale deployment in the automotive sector and for stationary applications. PEMFCs are characterized by several appealing features, including the following: (i) a high energy conversion efficiency in comparison to internal combustion engines (ICEs); (ii) a clean operation, whereby water is the only waste product; and (iii) a demonstrated suitability for introduction in a *smart* system of energy production, storage, and utilization.

The development of efficient low-temperature FCs is bottlenecked by the sluggish kinetics of the oxygen reduction reaction (ORR) that takes place at the cathode electrode. Therefore, intense research has been devoted to the design of highly efficient and durable ORR electrocatalysts (ECs). The features of an EC crucially contribute to the performance of a PEMFC; thus, the EC chemical composition, structure, and morphology must be carefully engineered to obtain devices that meet the requirements for practical applications. An optimal ORR EC must (i) minimize the ORR overpotential, typically by a high concentration of nanosized active sites characterized by a high turnover frequency and a uniform distribution; this results in a large electrochemically active surface area (ECSA); (ii) be a very good electrical conductor to minimize ohmic losses; (iii) possess a suitable morphology to promote the transport of reactants and products to and from the active sites; (iv) promote ion-exchange processes between the active sites and the ion-conducting membrane; (v) be stable under operating conditions to ensure good durability; and (vi) be synthesized through an easily scalable protocol to be compatible with large-scale industrial production.^2^

This Viewpoint discusses a new family of ECs exhibiting a hierarchical (H) *core–shell* support, wherein the *core* is covered by a carbon nitride (CN) *shell* stabilizing the active sites in unique *coordination nests*. The synthesis of the resulting *HCN-based ECs* is overviewed, highlighting the role played by each fundamental component and reaction step toward the design of ECs including Pt and exhibiting a well-controlled chemical composition, structure, and morphology to achieve a performance and durability level beyond the state of the art. The features of HCN-based ECs are correlated to the trends in performance observed both in *ex situ* studies via cyclic voltammetry with the thin-film rotating ring disk electrode (CV-TF-RRDE) method and *in situ* in a single PEMFC tested under operating conditions. Finally, the advantages of HCN-based ECs in comparison with state-of-the-art (SoA) ECs and with other families of ORR ECs with finely controlled morphologies are discussed, as well as the unique opportunities created by HCN-based ECs in marketable devices.

## 2 Main Families of ORR ECs

### 2.1 State-of-the-Art ECs

SoA ECs for the ORR exhibit highly dispersed Pt nanoparticles deposited on a conductive carbon support with a high surface area (Pt/C). Typical examples include ECs consisting of 40–50 wt % of Pt supported on Vulcan XC-72R carbon black. SoA ECs are generally synthesized through a modified polyol method, whereby a polyol (e.g., ethylene glycol) is oxidized while reducing metal ions to generate a metal colloid stabilized by the oxidized form of the polyol.^3^ This method provides ECs with Pt nanoparticles exhibiting a rather narrow size distribution (*d* ≈ 2–4 nm).

Nevertheless, the Pt loading provided by SoA ECs at PEMFC cathodes must be as high as 0.35 mg/cm^2^. Thus, up to 30 g of Pt are needed for a typical PEMFC stack powering a light-duty electric vehicle.^4^ This raises concerns in a high-volume production scenario because of the low abundance of Pt in Earth’s crust and the limited geographic distribution of Pt mines, leading to high costs and significant risks of supply bottlenecks. In this context, SoA Pt/C ECs still demonstrate an insufficient mass activity per unit mass of Pt (MA) and durability to meet the targets set by highly recognized institutes (e.g., the Department of Energy of the U.S. government, U.S. DOE) for high-performing, cost-competitive FC systems suitable for widespread applications.

The most accredited and popular strategy to design SoA ECs with a reduced Pt content and, at the same time, an enhanced ORR kinetics involves the introduction of a metal *co-catalyst* to form Pt–alloy nanostructures on carbon supports.^5^ Thus, Pt–alloy ECs including one or more metal co-catalyst such as Ni, Co, Fe, and lanthanides typically demonstrate a higher ORR activity than Pt/C ECs.^5,6^ In the last two decades, a very large variety of new Pt and Pt–alloy ECs has been reported in the scientific literature. Several of these ECs exhibit a MA that meets or exceeds the targets to achieve a widespread rollout of PEMFCs. However, such figures are often measured *ex situ* (e.g., through the thin-film rotating (ring) disk electrode, TF-R(R)DE, method). Hence, in this multitude of ECs it is difficult to identify actual alternatives to traditional SoA Pt/C ECs capable of combining a superior performance and durability with a scalable synthesis procedure that also allows the precise control of the features of the final material.

The traditional polyol method and its modifications, including sonication- and microwave-assisted processes, are largely employed for the synthesis of Pt/C and Pt–alloy ECs supported on a variety of carbon materials.^7^ Modified polyol methods enable the obtaining of ECs with a high loading of Pt and improved control over the distribution and size of Pt-based particles in comparison with other common synthesis methods based on wet impregnation followed by chemical reduction in the presence of a reducing agent or reduction under a hydrogen atmosphere at high temperature. Other common strategies to prepare Pt-based ECs consist of post-modification of commercial Pt/C ECs to introduce a co-catalyst and/or to dope the carbon support with heteroatoms.^8^ The formation of bimetallic catalysts can occur by deposition of the second metal onto Pt/C ECs, followed by annealing at high temperature, or by precipitation of the second metal as a hydroxide, or by incorporation of the second metal by sol–gel methods.^7^ However, these techniques provide limited control of particle size; particle growth and agglomeration are often observed.

### 2.2 Shape-Controlled ECs

The traditional methods, which are briefly overviewed in Section 2.1, do not allow for a precise control of the size, distribution, shape, and chemical composition of Pt/C and Pt–alloy ECs. This weak control of preparation processes of ECs curtails the kinetic performance, reducing the density of highly active species with low-index surface orientations of Pt and Pt alloys. In an attempt to address this issue, shape-controlled ECs were devised, featuring metal nanoparticles with specific shapes, such as cubes or octahedra, whereby particular surface terminations are preferentially exposed.^9^ These ECs, especially octahedrally shaped Pt–Ni ECs, are among the most active reported to date.^10^ The preparation of shape-controlled ECs typically uses wet-chemical synthesis procedures that involve organic high-boiling solvents, as well as surfactants and capping agents that are to be removed via post-synthesis treatments that often combine several washing, cleaning, and annealing steps.^9^ Therefore, the synthesis of shape-controlled ECs is generally complicated and time-consuming, and the use of organic solvents raises the costs in comparison to water-based or solid-state procedures. While some works reported the preparation of shape-controlled ECs in continuous flow reactors or with solid-state methods,^11^ the vast majority of shape-controlled ECs is obtained in small quantities and only tested *ex situ*; therefore, their performance in actual PEMFCs remains to be validated.^10^

### 2.3 HCN-based ECs

The HCN-based ECs here described were developed to obtain high-performing ECs also able to address the durability shortcomings of conventional Pt/C and especially Pt–alloy ECs. Indeed, Pt–alloy ECs including transition metal co-catalysts (see Section 2.1 and Section 2.2) tend to exhibit a poor durability owing to the occurrence of dealloying processes involving the preferential dissolution of the co-catalyst followed by Ostwald ripening and formation of large metal aggregates; at the same time, the carbon support is oxidized.^12^ Therefore, upon aging, in Pt–alloy ECs the utilization of Pt and the ECSA decrease, while ohmic losses increase. One effective strategy to stabilize the ORR EC as a whole is to create strong interactions between the metal particles bearing the active sites and the support. Such interactions must be able to inhibit (i) the leaching of metal species upon EC operation and (ii) the oxidation of the support. The introduction of nitrogen atoms in the carbon support is a highly promising strategy to achieve these goals, enhancing the coordination and stability of the active sites.^13^ Indeed, carbon nitride (CN)-based ECs bearing Pt and Pt-alloy active sites generally demonstrate an improved performance and durability in comparison with SoA Pt/C ECs because (i) N atoms promote the ORR kinetics through bifunctional and electronic effects, and (ii) the strong interactions mediated by the N atoms between the metals included in the active sites and the support inhibit the leaching of metal species, particle agglomeration, and support oxidation. The synthesis processes to obtain CN-based ECs also allow the tailoring of the features of the carbon supports, with a profound impact on the distribution, stability, and accessibility of the active sites.^14^ A broad variety of carbon materials, including carbon nanoparticles and nanotubes, graphene, and their derivatives have been extensively explored to shape the morphology of the final ECs.

Our group has developed an easily scalable synthesis procedure for the preparation of *core–shell* Pt-based ECs with a hierarchical (H) morphology, based on a carbon nitride (CN) matrix and including one or more co-catalysts. These *HCN-based ECs* possess a carbon *core* covered by a CN-based *shell*, upon which active sites are stabilized in *coordination nests* through strong interactions with C and N ligands. HCN-based ECs demonstrated a very promising performance and durability. The best HCN-based ECs include a very thin CN-based *shell* with a low concentration of N (<5 wt %) covering homogeneously the carbon *core*. N in this *shell* is located only in the nanoparticle *coordination nest*, i.e., in close proximity to the active sites. Accordingly, the thin layer of the CN *shell* is able to stabilize the metal alloy nanoparticle with the desired composition, structure, and shape, allowing to (i) maintain a facile charge transport between the external circuit and the active sites and (ii) obtain a good accessibility of reagents to the active site. The ohmic drops affecting the best HCN-based ECs are thus very similar to those introduced by conventional Pt/C and Pt–alloy ECs including only carbon supports. The proposed synthesis procedure has numerous favorable characteristics:

(a) Each reactant and process step serves to define well-understood aspects of the chemical composition, morphology, and structure of HCN-based ECs. Thus, these features can be modulated by rationally tuning the synthesis conditions.
(b) No sophisticated, highly specialized, and costly equipment is required.
(c) It easily provides HCN-based ECs at the gram scale, and it can be readily scaled-up.
(d) It can also be adapted to obtain high-performing platinum group metal (PGM)-free HCN-based ECs by replacing the Pt (or PGM) precursor with a non-PGM precursor.

This flexible yet simple procedure has been optimized over the years to obtain both PGM and PGM-free HCN-based ECs that approach or surpass the U.S. DOE targets for practical FCs.^15^ Furthermore, HCN-based ECs can be integrated into membrane–electrode assemblies (MEAs), the critical functional component at the heart of the PEMFC, through simple operations and without compromising their performance.

**Figure 1:**
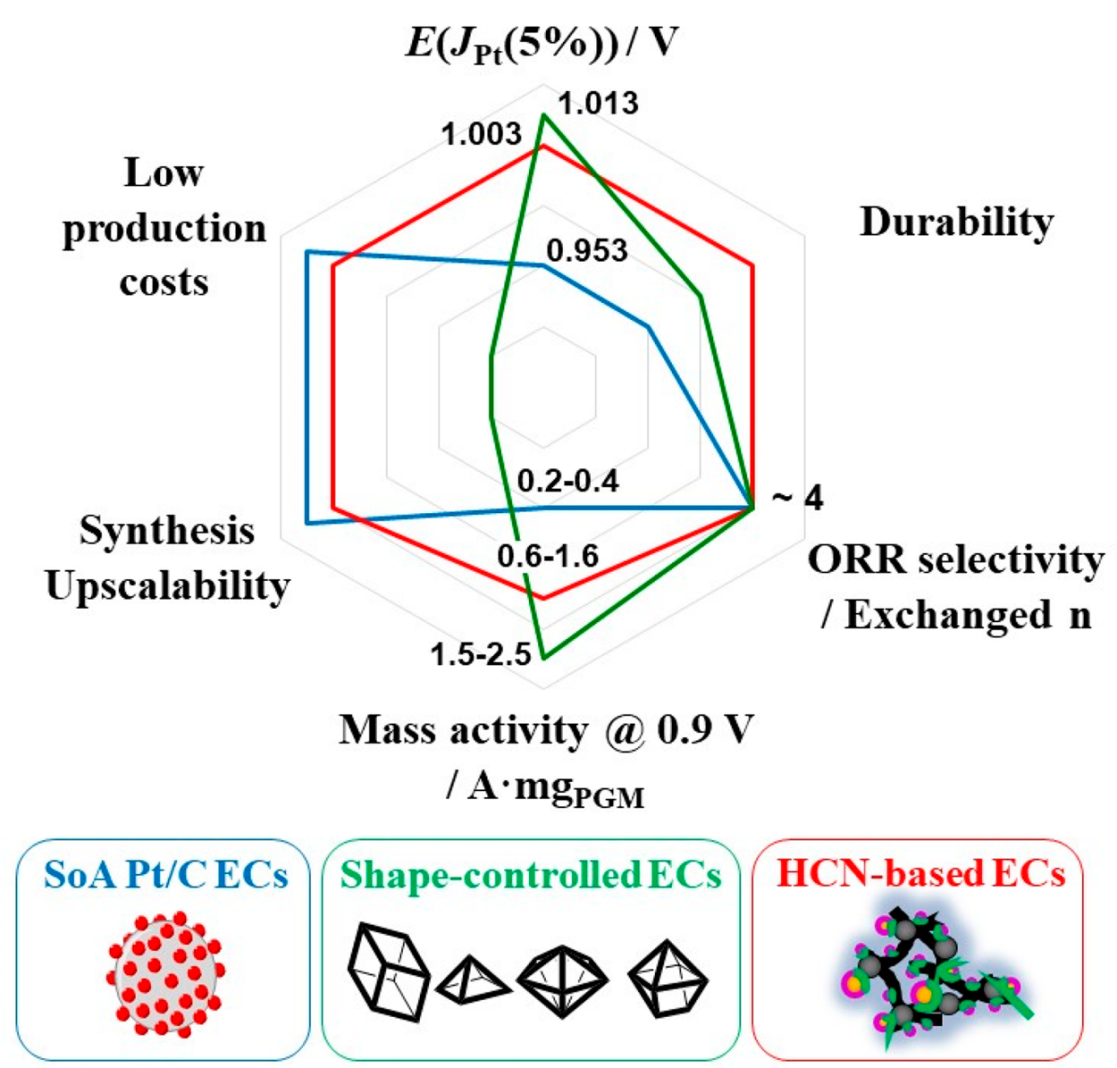
Comparison of relevant figures of merit for SoA ECs (blue line), shape-controlled ECs (green line), and HCN-based ECs (red line).

Figure 1 provides a general comparison between the most relevant figures of merit of (i) SoA ECs (both Pt/C ECs and Pt–alloy ECs), (ii) the HCN-based ECs developed in our laboratories, and (iii) shape-controlled ECs.

## 3 Desiderata for High-Performing PGM-Based ORR ECs

To foster the large-scale rollout of PEMFCs, it is paramount to develop PGM-based ECs that overcome the sluggish ORR kinetics, while also being highly durable, easy to produce in large amounts, and simple to implement in practical devices. The following Desiderata for PGM-based ORR ECs can then be identified:

(a) *Enhanced ORR kinetics at a relatively low PGM content.* The active sites must be devised to shift the onset potential of the ORR toward higher values (i.e., the activation barrier for the ORR, Δ*G*_ORR_^‡^, must be decreased) at a reduced PGM loading. High-performing ECs must exhibit a mass activity ≥0.44 A/mg_PGM_, measured at 0.9 V vs RHE, and achieve a specific power of 8 kW/g_PGM_ or more.^16,17^
(b) *High selectivity toward the four-electron ORR mechanism.* Indeed, the competing two-electron ORR mechanism yields H_2_O_2_, a highly oxidizing agent that is known to degrade the durability of the PEMFC.
(c) *Good charge transport*, which is strongly modulated by the electrical conductivity of the support.
(d) *Facile mass transport.* The ECs should exhibit a suitable morphology/porosity to promote the access of reactants to active sites. The ECSA of the EC should be at least on the order of 70–90 m^2^/g.^18^ The hydrophilicity/hydrophobicity of the support must allow for the removal of the water produced by the ORR, preventing flooding.
(e) *Long-term durability.* The active sites and the EC as a whole must remain stable during long-term FC operation. The latter should last for at least 8 000 h.^19^ The loss in mass activity should not exceed 40% of its initial value after 30 000 cycles.
(f) *Scalable synthesis*. The synthesis process should be simple, flexible, low-cost, and easy to upscale to facilitate the mass production of the EC.
(g) *Facile implementation in MEAs.* The ECs must be integrated in MEAs through a facile procedure that does not alter the intrinsic properties of the EC.

## 4 Features of PGM-Based ECs for the ORR That Meet the Desiderata

In order to meet the Desiderata for high-performing ORR ECs described in Section 3, the chemical composition, structure, morphology, and porosimetric features of PGM-based ECs must be carefully tailored, in compliance with the following points:

(a) Enhanced ORR kinetics and reduced PGM content are obtained by modulating the chemical composition of the active sites. The choice of the co-catalyst has a critical effect to raise the ORR onset potential and the turnover frequency of the EC. First-row transition metal co-catalysts such as Fe, Ni, and Co promote the ORR kinetics through electronic and bifunctional effects, e.g., promoting the first electron transfer from the EC to the incoming O_2_ molecule and favoring the desorption of the ORR products.^20^ The stoichiometric ratio between the PGM and co-catalyst is also important; a dealloying step is often necessary to remove the excess labile co-catalyst.^21^ In addition, the N atoms in the matrix, if present, contribute to enhancing the ORR kinetics through additional electronic/bifunctional effects.^2^ Finally, the size and uniformity of the active sites are critical to obtain a high PGM utilization.
(b) A high selectivity toward the 4e^–^ ORR mechanism is achieved by modulating the composition and stoichiometry of the active sites, as a high density of pairs of free neighboring PGM sites minimizes the yield of the H_2_O_2_ byproduct.^22^ The 4e^–^ selectivity is also raised by minimizing the presence of oxygen functionalities in the carbon matrix.
(c) Efficient charge transport depends on the choice of the carbon materials introduced in the support, as well as on the content of heteroatoms (e.g., N, S). Explored carbon materials for PGM-based ECs include Vulcan XC-72R carbon black, Ketjen Black, graphene, carbon nanotubes, and ordered meso/microporous carbons.^17^ Highly conductive carbon materials promote charge transport, and the synthesis procedure yielding the EC should ensure sufficient graphitization of the carbon matrix to further boost the conductivity.^2^ A high content of N atoms (>5 wt %) in CN-based ECs leads to high ohmic drops, which strongly impact the performance at intermediate/low voltages in an operating PEMFC. Therefore, it is preferred to locate N atoms only in close proximity to the active sites to enhance the activity and stability of the active sites without compromising the electrical conductivity of the EC.^2^
(d) Mass transport is facilitated by the presence of large, interconnected pores that provide an easy access to the incoming molecules of O_2_ reactant to the active sites. Therefore, the surface area of the EC and the distribution of macro-, meso-, and micropores play a fundamental role in the mass transport and in the accessibility of the active sites. Carbon materials with a high surface area (e.g., Ketjen Black) are generally beneficial for mass transport, resulting in an improved performance at intermediate/low potential in an operating PEMFC. HCN-based ECs can be designed to provide highly dispersed and accessible active sites.^20^ The morphology of the support may also affect the size of metal particles as it modulates the reaction conditions during the synthesis process; in turn, this may have a strong impact on the ECSA. Furthermore, a predominant distribution of metal particles on the outer surface of the carbon support can lead to ionomer poisoning of the EC.^17^ Thus, tuning the porosity features of the support and accommodating the active sites inside accessible pores are critical to preventing losses in EC performance.
(e) Durability is modulated by the stability of the active sites and of the support in the entire potential range covered by an operating PEMFC and under various back-pressures of feed gases. The establishment of strong interactions between the metal particles bearing the active sites and the support is beneficial to the stabilization of the EC. When the EC is integrated in the MEA, the presence of N atoms in the carbon-based support can raise the durability by promoting an improved ionomer distribution.^23^ It has also been demonstrated that pyrrolic nitrogen species inhibit carbon corrosion, thus leading to a higher and more stable performance.^24^ Moreover, the uniformity of metal particle size and dispersion onto the carbon support contribute to raising the durability and reducing the loss in mass activity and the corrosion of the carbon support. The latter is also influenced by the type and specific features of carbon materials (e.g., density of defects).^25^

Importantly, to close the gap between lab-scale research and industrialization, the following are critical:

(a) The synthesis of ECs is carried out through easily available and low-cost precursors and equipment, limiting the use of hazardous/polluting materials/solvents and time-consuming steps.
(b) The process to integrate the EC into the cathodic electrocatalytic layer and into the MEA is to be facile and scalable, and the conditions must be optimized to preserve the EC features.
(c) The MEA performance in PEMFCs must mirror as closely as possible the performance measured *ex situ* via CV-TF-RRDE. Though other techniques have become available to determine an EC performance under more practical conditions, such as the floating electrode techniques (FETs) and the half-cell gas diffusion electrode (GDE) method, single-cell MEA tests remain the most relevant and realistic.^10^

**Figure 2:**
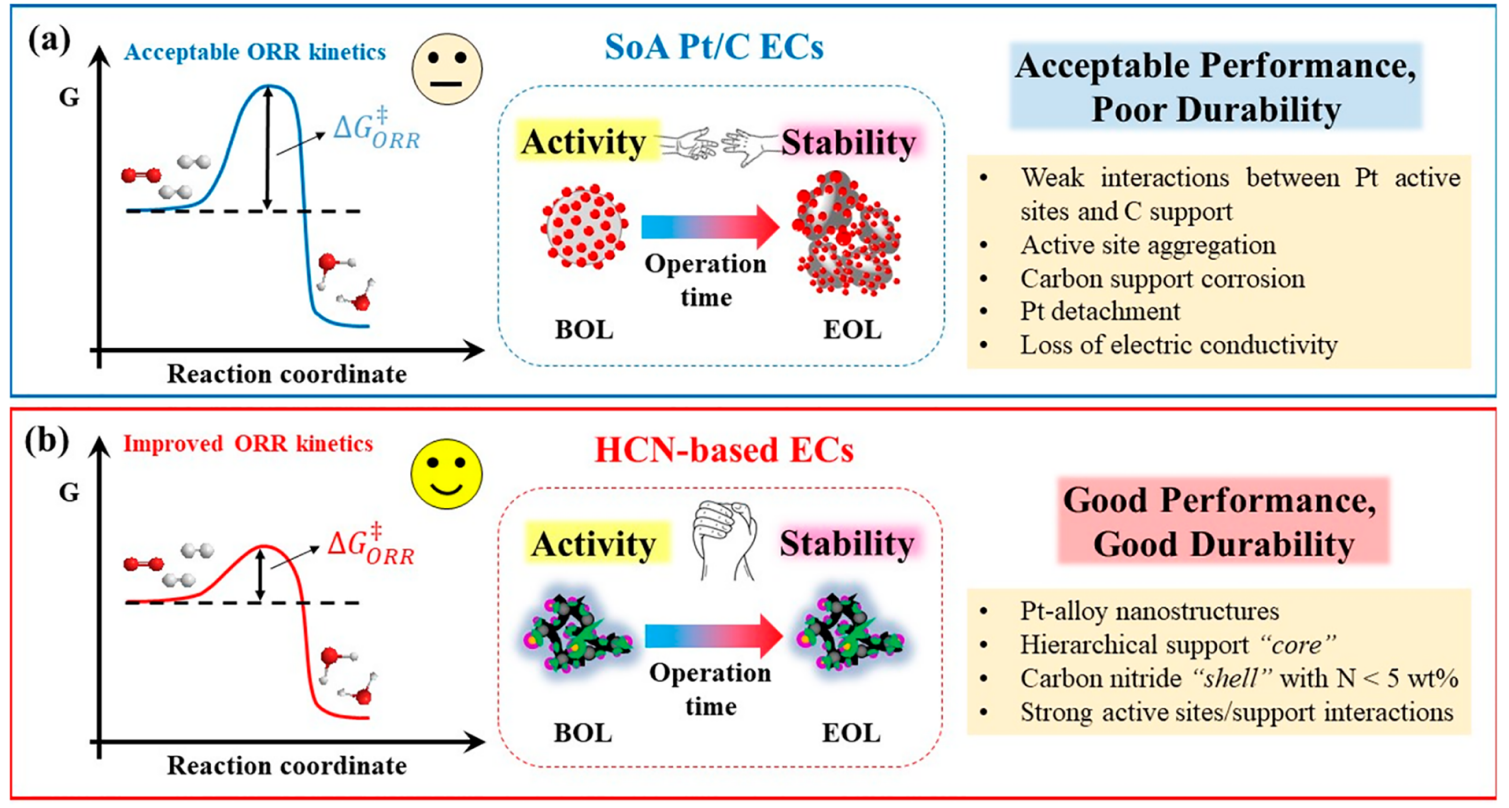
Simplified illustration of trends in energy diagrams of the ORR when employing (a) SoA Pt/C ECs and (b) HCN-based ECs (left). The general morphology of Pt/C ECs and HCN ECs at the beginning of life (BOL) and end of life (EOL) upon repeated cycling in PEMFCs is schematically shown in the center. The most important weaknesses of SoA Pt/C ECs and relevant strengths of HCN-based ECs are indicated on the right.

SoA Pt/C ECs without co-catalysts do not meet the Desiderata in terms of ORR kinetics and durability. The main reasons are attributed to the following (Figure 2a):

(a) the relatively large ORR overpotential (associated with a high activation energy barrier of the ORR, Δ*G*_ORR_) in comparison with most other advanced ECs bearing Pt–alloy active sites;
(b) the weak interactions between Pt nanoparticles and the carbon support, which result in metal dissolution and particle aggregation during prolonged cycling;
(c) the tendency of the carbon support to be corroded, further limiting the performance and durability.

Conversely, the features of HCN-based ECs are specifically tailored to meet the Desiderata and overcome the main weaknesses of SoA Pt/C ECs (Figure 2b). The next section elucidates how the various components and steps in the synthesis of HCN-based ECs cooperate to result in highly active and stable ORR catalysts.

## 5 Synthesis and Features of High-Performing HCN-Based ECs

**Figure 3:**
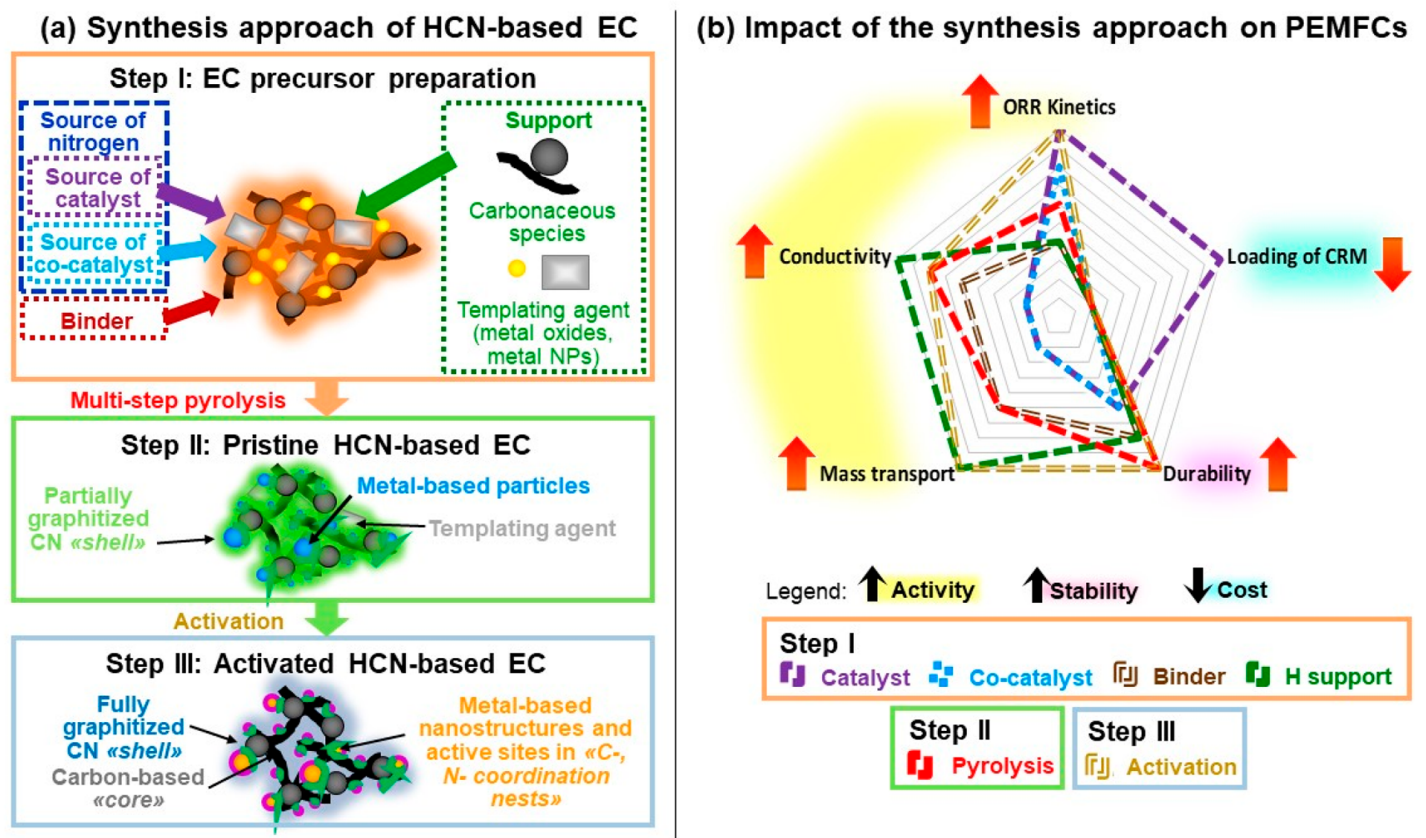
(a) Scheme of the general synthesis procedure for HCN-based ECs; (b) impact of the synthesis components and steps on the performance of HCN-based ECs in PEMFCs.

The synthesis of HCN-based ECs consists of three main steps (Figure 3a):

I. impregnation of the hierarchical support (H) with a zeolitic inorganic–organic polymer electrolyte (Z-IOPE);
II. multistep pyrolysis process;
III. activation process.

The following components are added during Step I: (i) the precursor of the *catalyst* (i.e., the element providing the largest contribution to the ORR kinetics; in HCN-based ECs it is typically Pt or Pd); (ii) the precursor(s) of the *co-catalyst(s)* (i.e., the element(s) boosting the ORR kinetics of the *catalyst*; in HCN-based ECs it is typically a first-row transition element such as Ni, Cu, Fe or Co); (iii) the binder, which is the main source of the C atoms of the CN *shell*; and (iv) the species forming the hierarchical support H. These components play a pivotal role in the determination of the composition, structure, and morphology of the final HCN-based EC. The precursor of the *catalyst*, the precursor(s) of the *co-catalyst(s)*, and the binder react forming a *zeolitic inorganic–organic polymer electrolyte—Z-IOPE*. The latter is a 3D-cross-linked system where metal atom complexes are embedded in anionic clusters bridged together by the molecules/macromolecules of the binder.^2,26−28^ In HCN-based ECs, the N atoms in the CN matrix (the *shell*) are typically provided by the cyano groups of the precursors of the co-catalyst(s). The selection of carbon materials for the support is flexible; different carbon types, including mixtures of different carbons, were used in reported HCN-based ECs.^29^ One or more *templating agents* can also be introduced during Step I. The introduction of a templating agent is meant to (i) improve the dispersion of the active sites on the EC surface; (ii) modulate the chemical composition of the active sites; and (iii) define the porosimetric features of the final EC. The templating agent must be removed (e.g., by chemical etching) during the subsequent synthesis steps to ensure that the charge and especially the mass transport features of the HCN-based ECs are optimized. The multistep pyrolysis process (Step II) begins with a low-temperature stage (*T* ≤ 300 °C) to consolidate the morphology and the structure of the system and form an *infusible* CN *shell* in the precursor. The following pyrolysis stages (i) trigger the nucleation and growth of the metal nanostructures bearing the active sites; (ii) graphitize the CN matrix on the support, raising the conductivity and durability; and (iii) create in the CN *shell* N- and C-*coordination nests* that stabilize the above-mentioned metal nanostructures.^2,29,30^ In Step III the EC activation is carried out by different procedures that may involve both chemical and/or electrochemical treatments. The latter are meant to etch only undesired species found mostly in the surface of the system. Step III eliminates excess co-catalyst and further modulates the composition, structure, and morphology of the final *shell* of the HCN-based EC (e.g., removing labile synthesis byproducts). The following paragraphs present the synthesis protocol of HCN-based ECs in more detail and discuss the impact of each component and step on the final performance, which is summarized in Figure 3b.

### 5.1 *Catalyst* and *Co-catalyst* Precursors as Nitrogen Sources

In HCN-based ECs, the catalyst and co-catalyst precursors provide the following: (i) the metal species forming the ORR active sites and (ii) the N atoms that will be included in the carbon nitride (CN) matrix (the *shell*). At the beginning of our research on ECs, our interest was focused on materials based only on the CN matrix and without a carbon *core*. To obtain these ECs, we used macromolecules containing N (e.g., polyacrylonitrile)^28^ both (i) as the source of N atoms and (ii) as ligands to obtain a hybrid inorganic–organic precursor for the final EC. At the end of the synthesis process, the EC consisted of a CN matrix dispersing homogeneously, both on the surface and in the bulk, a high concentration of N atoms (>5 wt %). This led to an EC triggering a high ohmic drop, though a good tolerance to ORR contaminants was also achieved.^2,26,28^ Further studies demonstrated that ECs based on a CN matrix that included a limited amount of N atoms (<5 wt %) located mostly around the metal particles bearing the active sites exhibited an improved ORR kinetics and yielded a lower ohmic drop. These ECs were obtained by pyrolyzing a precursor based on a Z-IOPE. The CN matrix of the resulting EC consists of N- and C-*coordination nests*, which stabilize the metal nanostructures bearing the active sites by forming strong covalent interactions. In a typical protocol for the preparation of Pt–alloy HCN-based ECs, the catalyst precursor is a metal complex bearing good leaving groups, e.g., a chlorometalate compound such as K_2_PtCl_4_. The co-catalyst precursor is a cyanometalate compound, e.g., K_2_Ni(CN)_4_ or K_4_Fe(CN)_6_. This reaction scheme is very flexible, as it can be implemented with a large variety of K_*a*_M_1_Cl_*b*_ and K_*c*_M_*j*_(CN)_*k*_ compounds where M_1_ and M_*j*_ can be either PGMs (e.g., Pt, Pd, Rh, ...) or non-PGMs (e.g., Sn, Fe, Co, Ni, ...). Hence, the proposed reaction scheme can yield a *PGM-free* Z-IOPE; the latter can be used in the synthesis of PGM-free HCN-based ECs.^15,31^ Other heteroatoms (e.g., S) can be introduced in the HCN-based EC by using catalyst and/or co-catalyst precursors bearing suitable ligands (e.g., SCN). The selection of metal precursors has a very important impact on the implementation of HCN-based ECs in a PEMFC and on the performance of the device (Figure 3b). In conclusion, the selected metals and their stoichiometry affect the following: (i) the loading of critical raw materials (e.g., Pt) in the final HCN-based ECs and thus the cost of the ECs and of the final PEMFC stack; (ii) the ORR kinetics; and (iii) to some extent, the durability of the final HCN-based EC (e.g., typically, the ECs including Cu as a co-catalyst present a lower durability with respect to those based on Ni).

### 5.2 Binder and Z-IOPE Formation

**Figure 4:**
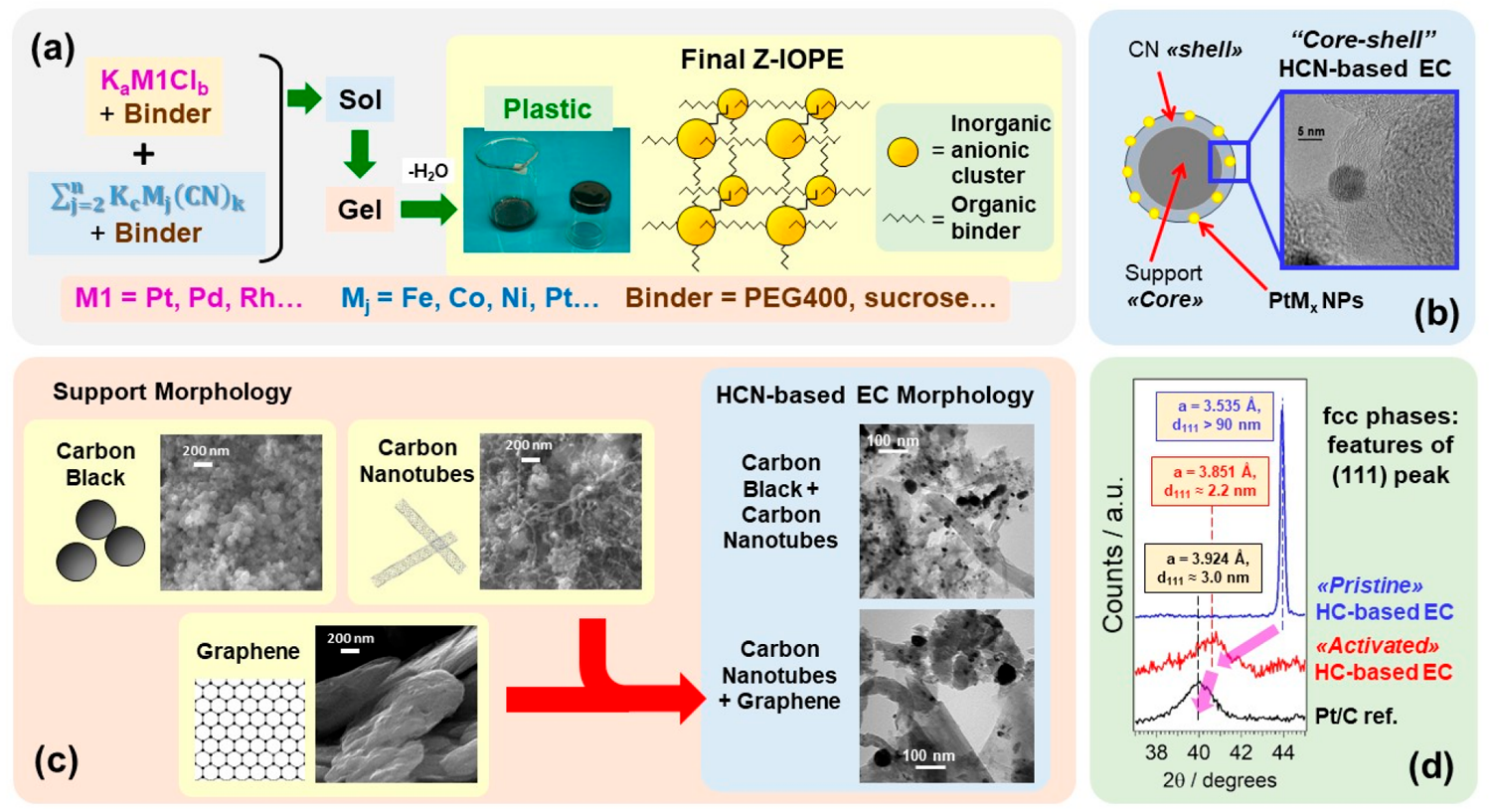
Selected features of HCN-based ECs: (a) details of the Z-IOPE synthesis; (b) typical *core–shell* morphology of HCN-based ECs (figure reproduced with permission from The Electrochemical Society);^2^ (c) impact of the morphology of the support on that of the final HCN-based ECs; and (d) example of the effect of the activation process on the structural features of the fcc phases of HCN-based ECs.

The Z-IOPE is typically obtained by reacting the following: (i) an aqueous solution including the chlorometalate complex and the binder and (ii) a second aqueous solution including the cyanometalate specie(s) and the binder (Figure 4a). The binder is typically an organic molecule/macromolecule with a high density of −OH groups such as sucrose or poly(ethylene oxide).^26^ In a complex series of chemical equilibria (i) the cyanometalate ligand coordinates the metal of the chlorometalate complex by displacing its chloride groups, forming clusters of complexes bridged by −CN– groups, and (ii) these clusters are then networked by the binder, triggering a sol → gel and gel → plastic transition that yields the final Z-IOPE (Figure 4a). The precursor of the HCN-based ECs is obtained as follows. (i) The solid species forming the H support and, optionally, the *templating agent* are added to the aqueous solutions used to synthesize the Z-IOPE. (ii) The chlorometalate, the cyanometalate complex(es), and the binder adsorb on the surface of the solid species giving rise to the H support. (iii) The above-described sol → gel and gel → plastic transition forms a thin layer of Z-IOPE precursor wrapping the surface of the solid H support. The type and amount of binder used affect the uniformity and thickness of the Z-IOPE layer covering the H support of the HCN-based EC precursor. These parameters are crucial to the modulation of the uniformity and thickness of the CN matrix forming the homogeneous *shell* of the H support in the final HCN-based ECs. This thin, uniform CN *shell* stabilizes the metal alloy nanoparticles bearing the active sites in N- and C-*coordination nests*. In conclusion, the binder has a modest impact on the charge and mass transport properties (Figure 3b) and a significant impact on the consolidation of the active sites in the *coordination nests* and thus on the durability of HCN-based ECs.

### 5.3 H Support: Carbonaceous Species and Templating Agent(s)

The H support can be obtained starting from a wide variety of carbon materials. The latter are selected to bestow the final HCN-based EC beneficial properties including the following: (i) a high electrical conductivity, to minimize ohmic losses; (ii) a large surface area, to facilitate mass transport; and (iii) a good tolerance to oxidizing conditions, to raise durability.^32^ Carbon black is frequently used owing to the large surface area and the low microporosity, as well as the possibility to easily control the surface concentration of −OH groups, modulating the hydrophilicity. However, carbon black limits the possibility to fine-tune the pore structure of the final EC. Furthermore, other carbon materials (e.g., graphene and carbon nanotubes) exhibit a higher electrical conductivity than carbon black. Graphene is characterized by a high electrical conductivity and by a very low microporosity that raises the accessibility of the active sites. These features prompted the synthesis of HCN-based ECs wherein the H support includes graphene and other carbon materials. Graphene derivatives such as graphene oxide (GO) and reduced GO (RGO) can also be used in the H support. Carbon nanotubes (CNTs) exhibit a good electrical conductivity and thermal stability and were also used in the synthesis of HCN-based ECs. The presence of defects such as edges and/or heteroatoms on graphene, CNTs, and their derivatives facilitates the nucleation of the metal nanostructures bearing the active sites during the synthesis of the HCN-based EC. This affects the distribution of the active sites on the CN *shell* and modulates the structure and interactions of the CN *shell* and of the H support. During Step I of the HCN-based EC synthesis, the selected carbon materials that form the H support are wrapped by the Z-IOPE.^2^ Thus, after the synthesis the carbon materials end up composing the conductive *core* wrapped by the CN 3D network constituting the *shell* (Figure 4b). The weight ratio between (i) the carbon materials used to obtain the H support and (ii) the *catalyst* and *co-catalyst(s)* precursors determines the metal content in the final HCN-based EC. The properties of the carbon materials affect strongly the morphology and the porosity features of the final HCN-based ECs (Figure 4c). In conclusion, the H support has a critical impact on the mass transport features and the electrical conductivity of the HCN-based ECs (Figure 3b). The hydrophilicity of the H support determines the polarity of the surface of the final ECs and thus the extent of their flooding during cycling in a FC; this modulates the durability of the HCN-based EC. Finally, the porosity features of the H support have a strong impact on reagent accessibility to the active sites of ECs.

The use of a hard template is a common strategy presented in the literature to synthesize porous carbon supports with an exceptionally high surface area and well-controlled porosity features.^33^ In the synthesis of HCN-based ECs, the templating agent is a sacrificial component introduced in the H support. The templating agent modulates the morphology of both the H support and of the entire HCN-based EC by constraining the diffusion of both the organic species and the metals during the pyrolysis process. As a result, it is possible to modulate the size and the dispersion of the metal nanostructures that will bear the active sites for the ORR. In some instances (e.g., using templating agents consisting of first-row transition metals and first-row transition metal oxides such as Cu and CuO_*x*_), during the pyrolysis process the atoms of the templating agent diffuse into the metal nanostructures that will bear the active sites for the ORR. This affects the ORR kinetics of the final HCN-based EC. The templating agent is etched before the final HCN-based EC is obtained. The chemical composition of the templating agent is selected considering the following: (i) how easily it can be mixed with carbon materials, to obtain a homogeneous precursor of the HCN-based EC; (ii) how stable it is at the high temperatures of the pyrolysis process, to minimize undesired side reactions; and (iii) how easily it can be etched without damaging the performance and durability of the final HCN-based EC. Metal oxide nanoparticles are common templating agents and include (i) SiO_2_ (suitable to obtain a mesoporous support), (ii) MgO, and (iii) ZnO. ZnO is unstable at *T* > 500 °C, as it reacts with carbon species; thus, it can possibly affect negatively the morphology of the final system.^32^ The effect of the templating agent on the features of HCN-based ECs is included in the impact of the support in Figure 3b. Specifically, in HCN-based ECs the templating agent plays a major role to (i) raise the surface area, (ii) define the porosity, and (iii) modulate the distribution and accessibility of the active sites. To a lesser extent, the templating agent may affect the chemical composition of the active sites for the ORR. In conclusion, the templating agent has a strong impact on mass transport properties and a minor impact on the ORR kinetics of the HCN-based ECs.

### 5.4 Multistep Pyrolysis

The multistep pyrolysis process in an inert atmosphere or under vacuum yields HCN-based ECs with a well-defined *core–shell* morphology and active sites stabilized in C- and N-*coordination nests*. The first pyrolysis step is carried out at a relatively low temperature (150–300 °C) to (i) eliminate water and other low-molecular weight compounds and (ii) consolidate the morphology and the structure of the CN *shell*. Thus, an infusible precursor of the *shell* is obtained, wherein metal atoms are still homogeneously dispersed in the CN matrix.^2,33^ The following pyrolysis step(s) are conducted at higher temperature (400–900 °C). During the high-temperature pyrolysis, metal atoms diffuse through the CN matrix, and the metal nanoparticles/nanostructures bearing the ORR active sites nucleate and grow in this phase.^2,29^ Correspondingly, the metal nanoparticles/nanostructures establish strong interactions with the CN *shell* via N- and C-ligands of *coordination nests*, which are critical to achieving a high durability (Figure 3a).^22,27^ The high temperature also promotes the graphitization of the CN matrix, raising the conductivity of the HCN-based EC.^2^ The thermal stability of the templating agent, if introduced in Step I, must be considered when establishing the pyrolysis temperature. This is to prevent undesired decomposition phenomena that could be detrimental to the performance and the durability of the HCN-based ECs. In conclusion (Figure 3b), the pyrolysis step has a moderate impact (i) on the ORR kinetics, as it rearranges metal atoms that are already present in the system to form the active sites, and (ii) on the mass transport properties, as it modulates the morphology of the metal nanoparticles/nanostructures bearing the active sites. The pyrolysis step has a strong impact on charge transport, as it controls the progress of the graphitization of the CN *shell*. Finally, the pyrolysis step has a crucial impact on durability because it establishes strong interactions between the active sites on metal alloys and the CN *shell* of the HCN-based ECs.

### 5.5 Activation

The final step in the synthesis of HCN-based ECs is an activation process, which (i) eliminates labile reaction byproducts, (ii) removes excess *co-catalyst(s)*, and (iii) stabilizes the *catalyst* (typically a PGM such as Pt or Pd) nanoparticles as much as possible. The elimination of labile reaction byproducts (i) removes contaminants from the active sites, raising ORR kinetics and accessibility; and (ii) improves the graphitization of the CN matrix, improving electrical conductivity and durability. The process that removes the excess co-catalyst(s) and raises the stoichiometric ratio between catalyst and co-catalyst is better known as *“dealloying”*. Upon activation, (i) the stoichiometry of the active sites is fixed, and (ii) the exposure of the active sites to the environment is raised (Figure 4b and c), improving accessibility and ECSA. The dealloying process is driven by the Kirkendall effect,^34^ a vacancy-mediated diffusion mechanism whereby the co-catalyst(s) atoms diffuse faster than Pt and are eventually removed, resulting in a modification of the chemical composition and morphology of the metal nanoparticles/nanostructures bearing the active sites. Dealloying affects the interatomic distance between Pt atoms in the fcc PtM_*x*_ alloys that typically bear the active sites in HCN-based ECs, triggering electronic effects that raise the ORR kinetics (Figure 4d).^21^ Dealloying can also decrease the grain size of the same alloys (Figure 4d). The activation/dealloying process can be achieved through either/both chemical and electrochemical processes (e.g., by multiple treatments of the EC with a diluted acid solution). In conclusion, the final activation step has a massive impact on ORR kinetics, electrical conductivity, mass transport features, and durability of the HCN-based ECs (Figure 3b) and is a unique and crucial aspect that distinguishes the proposed synthesis route from all the other methods described in the literature.

## 6 Selected Examples on the Impact of HCN-Based EC Features on Their Performance and Durability

**Figure 5:**
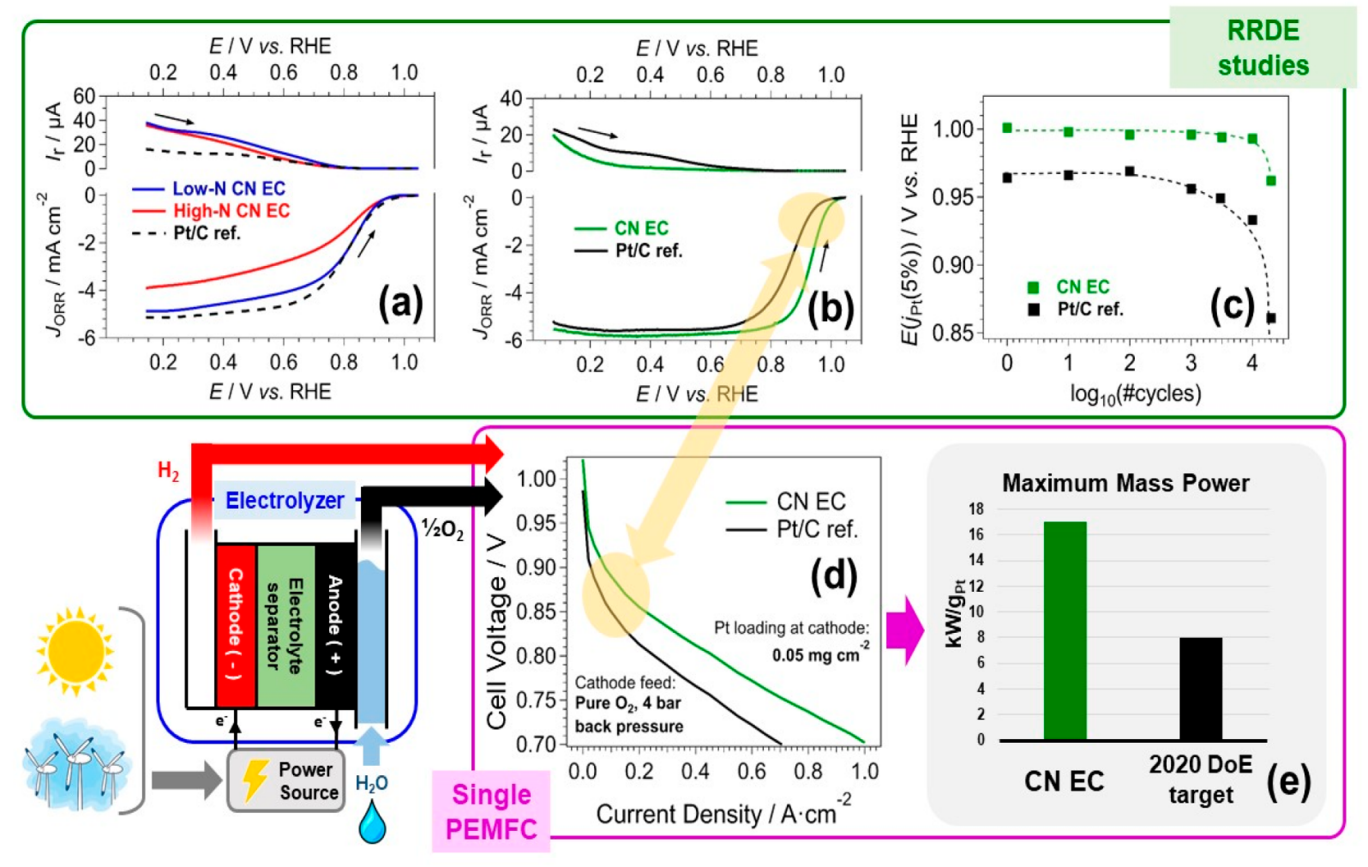
Comparison between the performance of SoA Pt/C ECs and HCN-based ECs measured via CV-TF-RRDE (top) and in a single PEMFC under operating conditions (bottom). The data show the impact of (a) N content in HCN-based ECs: high-N EC (red line); low-N EC (blue line); Pt/C reference (black, dashed line) (data reproduced with permission from Wiley);^35^ (b, c, d, e) introduction of Ni co-catalyst in an HCN-based EC including a CN *shell* matrix with N < 5 wt % (CN EC, marked in green) using a SoA Pt/C EC as the reference (Pt/C ref., marked in black). Effect on the following: (b) ORR profiles; (c) durability; (d) MEA performance; (e) maximum mass power in a single PEMFC relative to the 2020 U.S. DOE target.^16^

The synthesis strategy yielding HCN-based ECs has been tailored to design ECs with continually improving features and able to meet the performance targets set by international organizations such as the U.S. DOE. The impact of the N content was observed in the early stages of these studies and represented a breakthrough in the development of HCN-based ECs.^2^Figure 5a shows the ORR profiles (measured via the CV-TF-RRDE method in an acidic environment) of two trimetallic ECs including active sites composed of Pd, Co, and Ni embedded in a CN matrix with a N content either >5 wt % (high-N) or <5 wt % (low-N), compared with a SoA Pt/C EC.^35^ The high-N EC shows much poorer performance at intermediate and low potentials, indicative of a lower electrical conductivity and a less effective mass transport. These characteristics are largely improved when N atoms are introduced in limited amount and only located in the proximity of the active sites. Moreover, the onset potential measured as *E*(*J*_Pt_(5%))^36^ increases from high-N (0.907 V) to low-N (0.917 V), approaching the value exhibited by the Pt/C ref. (0.932 V). This suggests that the presence of N atoms close to the active sites contributes to enhancing of the ORR kinetics through bifunctional/electronic effects. The selectivity of the ECs for the 4e^–^ reduction mechanism, which can be inferred from the current collected on the ring electrode, is unaffected by the N content and is lower than that of the SoA EC, owing to mainly the presence of oxophilic Pd in the active sites.^35^

Both the ORR kinetics and selectivity in the 4e^–^ mechanism were then strongly improved by designing Pt–alloy HCN-based ECs with N < 5 wt %, using Ni as a co-catalyst (Figure 5b). Ni behaves as a strong Lewis acid and promotes the desorption of the ORR reaction products via a bifunctional mechanism. In addition, the ORR kinetics is improved by electronic effects triggered by the compression of the cell constant of Pt upon alloying with Ni. As a consequence, the *E*(*J*_Pt_(5%)) of the HCN-based EC is ca. 40 mV more positive than the *E*(*J*_Pt_(5%)) of the Pt/C EC, and the ring current is slightly decreased (corresponding to a lower yield of H_2_O_2_). The PtNi HCN-based EC demonstrated much improved durability in comparison with the Pt/C EC in CV-TF-RRDE tests (Figure 5c), with the ORR overpotential increasing by only ca. 35 mV upon 20 000 accelerated aging cycles, in contrast with a >100 mV increase for the SoA EC in the same conditions.

The effective translation of EC performance from the RRDE setting to the MEA and the FC device remains the most important challenge toward the development and commercialization of novel ORR ECs; this is particularly evident for shape-controlled ECs.^10^ Conversely, HCN-based ECs can be integrated into MEAs without compromising their features, obtaining single PEMFC performance that mirrors quite closely the outcome of CV-TF-RRDE studies (Figure 5b and d).

Figure 5d shows the polarization curve of the PtNi HCN-based EC described above, measured using pure oxygen as the fuel and under a high back-pressure of 4 bar. The Pt loading in the cathodic electrocatalytic layer of the MEA is 0.05 mg_Pt_/cm^2^. Under these conditions, with respect to the Pt/C benchmark, the *E*(*J*_Pt_(5%)) of the HCN-based EC is positively shifted by ca. 40 mV as in the CV-TF-RRDE study. Considering the low Pt content at the cathode, the MEA mounting the HCN-based EC reaches a maximum mass power of ca. 17 kW/g_Pt_, which is more than twice the 2020 U.S. DOE target.^16^

HCN-based ECs tend to exhibit an improved performance in a single PEMFC, especially under a high back-pressure of reactant gases and in particular when pure O_2_ is used as the oxidant. This opens the door to a yet-unexplored concept in FC technologies. Indeed, the urgency and continuous investments to realize a *green* hydrogen economy are driving the rapid development and commercialization of electrolyzers (ELs) that produce *green* H_2_ from water using electricity obtained from renewable sources. Though the H_2_ production currently attracts the most attention owing to its use in FCs, industrial processes, etc., it should be noted that the water electrolysis also yields pure O_2_. However, this latter gas is typically dumped in the atmosphere with a significant loss of process efficiency. This wasteful approach is justified if we consider that when conventional SoA Pt/C ECs operate under pure O_2_, they do not exhibit a durability level complying with the requirements set by applications. Thus, O_2_ is virtually useless as an oxidant for PEMFCs meant for most conventional applications. On the other hand, HCN-based ECs are expected to be much more tolerant to oxidative degradation owing to (i) the resilience of the CN *shell* and (ii) the strong interactions between the CN *shell* and the metal nanoparticles/nanostructures bearing the ORR active sites. This would result in a performance level that surpasses that afforded by SoA ECs (Figure 5d) with an adequate durability, at the same time preventing the waste of the O_2_ produced in ELs.

## 7 Conclusion and Perspectives

HCN-based ECs represent a new and very promising family of high-performing ORR ECs that exhibit improved ORR kinetics and durability in comparison with SoA ECs as a result of the rational tailoring of their chemical composition, structure, and morphology. Moreover, HCN-based ECs are prepared through an extremely flexible and easily scalable synthesis protocol, and they demonstrate comparable performance in CV-TF-RRDE studies and in a single PEMFC. This is in stark contrast with a multitude of exotic ECs reported in the literature (e.g., Pt alloys, shape-controlled systems) that display outstanding performance in CV-TF-RRDE studies only. In addition, HCN-based ECs are still similar enough to SoA ECs to be able to exploit the technical advancements in other MEA components (e.g., ion-exchange membrane, gas-diffusion layers). Therefore, HCN-based ECs hold great promise as next-generation ECs for PEMFCs with a lower cost, better performance, and durability and very suitable for large-scale rollout.

Furthermore, and very differently from SoA ECs, the unique features and the strong interactions between the metal nanoparticles/nanostructures bearing the active sites and the CN-based *shell* covering the support of HCN-based ECs likely make these latter materials suitable for long-term operation under a high back-pressure of pure O_2_ used as the oxidant. This hints at the possibility to usefully exploit not only the *“green hydrogen”* that is produced in a water EL but also the O_2_ that is obtained simultaneously and that would otherwise be wasted. It would only be necessary to feed directly both gases into the PEMFC mounting the HCN-based ECs at the cathode. Therefore, HCN-based ECs could open a new perspective whereby it may become possible to take full advantage of the fast growth of green hydrogen production to reap additional benefits from the large-scale implementation of the hydrogen economy.

## References

[ref1] Atanassov P.; Di Noto V.; McPhail S. From Hydrogen Manifesto, through Green Deal and Just Transition, to Clean Energy Act. Electrochem. Soc. Interface 2021, 30 (4), 57 10.1149/2.F14214IF.

[ref2] Di Noto V.; Negro E.; Vezzù K.; Bertasi F.; Nawn G. Origins, developments, and perspectives of carbon nitride-based electrocatalysts for application in low-temperature FCs. Electrochemical Society Interface 2015, 24 (2), 59 10.1149/2.F05152if.

[ref3] Esmaeilifar A.; Rowshanzamir S.; Eikani M.; Ghazanfari E. Synthesis methods of low-Pt-loading electrocatalysts for proton exchange membrane fuel cell systems. Energy 2010, 35 (9), 3941–3957. 10.1016/j.energy.2010.06.006.

[ref4] Pollet B. G.; Kocha S. S.; Staffell I. Current status of automotive fuel cells for sustainable transport. Current opinion in Electrochemistry 2019, 16, 90–95. 10.1016/j.coelec.2019.04.021.

[ref5] Kulkarni A.; Siahrostami S.; Patel A.; Nørskov J. K. Understanding catalytic activity trends in the oxygen reduction reaction. Chem. Rev. 2018, 118 (5), 2302–2312. 10.1021/acs.chemrev.7b00488. 29405702

[ref6] Escudero-Escribano M.; Malacrida P.; Hansen M. H.; Vej-Hansen U. G.; Velázquez-Palenzuela A.; Tripkovic V.; Schiøtz J.; Rossmeisl J.; Stephens I. E.; Chorkendorff I. Tuning the activity of Pt alloy electrocatalysts by means of the lanthanide contraction. Science 2016, 352 (6281), 73–76. 10.1126/science.aad8892. 27034369

[ref7] Santiago E. I.; Varanda L. C.; Villullas H. M. Carbon-supported Pt– Co catalysts prepared by a modified polyol process as cathodes for pem fuel cells. J. Phys. Chem. C 2007, 111 (7), 3146–3151. 10.1021/jp0670081.

[ref8] Corpuz A. R.; Wood K. N.; Pylypenko S.; Dameron A. A.; Joghee P.; Olson T. S.; Bender G.; Dinh H. N.; Gennett T.; Richards R. M.; et al. Effect of nitrogen post-doping on a commercial platinum–ruthenium/carbon anode catalyst. J. Power Sources 2014, 248, 296–306. 10.1016/j.jpowsour.2013.09.067.

[ref9] Beermann V.; Gocyla M.; Kühl S.; Padgett E.; Schmies H.; Goerlin M.; Erini N.; Shviro M.; Heggen M.; Dunin-Borkowski R. E.; et al. Tuning the electrocatalytic oxygen reduction reaction activity and stability of shape-controlled Pt–Ni nanoparticles by thermal annealing– elucidating the surface atomic structural and compositional changes. J. Am. Chem. Soc. 2017, 139 (46), 16536–16547. 10.1021/jacs.7b06846. 29019692

[ref10] Pan L.; Ott S.; Dionigi F.; Strasser P. Current challenges related to the deployment of shape-controlled Pt alloy oxygen reduction reaction nanocatalysts into low Pt-loaded cathode layers of proton exchange membrane fuel cells. Current Opinion in Electrochemistry 2019, 18, 61–71. 10.1016/j.coelec.2019.10.011.

[ref11] Niu G.; Zhou M.; Yang X.; Park J.; Lu N.; Wang J.; Kim M. J.; Wang L.; Xia Y. Synthesis of Pt–Ni octahedra in continuous-flow droplet reactors for the scalable production of highly active catalysts toward oxygen reduction. Nano Lett. 2016, 16 (6), 3850–3857. 10.1021/acs.nanolett.6b01340. 27135156

[ref12] Dubau L.; Lopez-Haro M.; Castanheira L.; Durst J.; Chatenet M.; Bayle-Guillemaud P.; Guétaz L.; Caqué N.; Rossinot E.; Maillard F. Probing the structure, the composition and the ORR activity of Pt3Co/C nanocrystallites during a 3422 h PEMFC ageing test. Appl. Catal. B: Environmental 2013, 142, 801–808. 10.1016/j.apcatb.2013.06.011.

[ref13] Singh S. K.; Takeyasu K.; Nakamura J. Active sites and mechanism of oxygen reduction reaction electrocatalysis on nitrogen-doped carbon materials. Adv. Mater. 2019, 31 (13), 1804297 10.1002/adma.201804297. 30350433

[ref14] Gerber I. C.; Serp P. A theory/experience description of support effects in carbon-supported catalysts. Chem. Rev. 2020, 120 (2), 1250–1349. 10.1021/acs.chemrev.9b00209. 31577439

[ref15] Negro E.; Bach Delpeuch A.; Vezzù K.; Nawn G.; Bertasi F.; Ansaldo A.; Pellegrini V.; Dembinska B.; Zoladek S.; Miecznikowski K.; et al. Toward Pt-Free Anion-Exchange Membrane Fuel Cells: Fe–Sn Carbon Nitride–Graphene Core–Shell Electrocatalysts for the Oxygen Reduction Reaction. Chem. Mater. 2018, 30 (8), 2651–2659. 10.1021/acs.chemmater.7b05323.

[ref16] Fuel Cell Technologies Office Multi-Year Research, Development, and Demonstration Plan-Section 3.4 Fuel Cells; 2016 (Updated May 2017). https://www.energy.gov/sites/default/files/2017/05/f34/fcto_myrdd_fuel_cells.pdf.

[ref17] Banham D.; Zou J.; Mukerjee S.; Liu Z.; Yang D.; Zhang Y.; Peng Y.; Dong A. Ultralow platinum loading proton exchange membrane fuel cells: Performance losses and solutions. J. Power Sources 2021, 490, 229515 10.1016/j.jpowsour.2021.229515.

[ref18] Gasteiger H. A.; Kocha S. S.; Sompalli B.; Wagner F. T. Activity benchmarks and requirements for Pt, Pt–alloy, and non-Pt oxygen reduction catalysts for PEMFCs. Appl. Catal. B: Environmental 2005, 56 (1), 9–35. 10.1016/j.apcatb.2004.06.021.

[ref19] Papageorgopoulos D. U.S. Department of Energy (DOE) Hydrogen Program 2021 Annual Merit Review Proceedings; 2021. https://www.hydrogen.energy.gov/pdfs/review21/plenary8_papageorgopoulos_2021_o.pdf.

[ref20] Di Noto V.; Negro E. Pt–Fe and Pt–Ni Carbon Nitride-Based ‘Core–Shell’ ORR Electrocatalysts for Polymer Electrolyte Membrane Fuel Cells. Fuel Cells 2010, 10 (2), 234–244. 10.1002/fuce.200900129.

[ref21] Koh S.; Strasser P. Electrocatalysis on bimetallic surfaces: modifying catalytic reactivity for oxygen reduction by voltammetric surface dealloying. J. Am. Chem. Soc. 2007, 129 (42), 12624–12625. 10.1021/ja0742784. 17910452

[ref22] Di Noto V.; Negro E.; Polizzi S.; Agresti F.; Giffin G. A. Synthesis–Structure–Morphology Interplay of Bimetallic “Core–Shell” Carbon Nitride Nano-electrocatalysts. ChemSusChem 2012, 5 (12), 2451–2459. 10.1002/cssc.201200517. 23019172

[ref23] Hornberger E.; Merzdorf T.; Schmies H.; Hübner J.; Klingenhof M.; Gernert U.; Kroschel M.; Anke B. r.; Lerch M.; Schmidt J.; et al. Impact of Carbon N-Doping and Pyridinic-N Content on the Fuel Cell Performance and Durability of Carbon-Supported Pt Nanoparticle Catalysts. ACS Appl. Mater. Interfaces 2022, 14 (16), 18420–18430. 10.1021/acsami.2c00762. 35417125

[ref24] Schmies H.; Hornberger E.; Anke B. r.; Jurzinsky T.; Nong H. N.; Dionigi F.; Kühl S.; Drnec J.; Lerch M.; Cremers C.; et al. Impact of carbon support functionalization on the electrochemical stability of Pt fuel cell catalysts. Chem. Mater. 2018, 30 (20), 7287–7295. 10.1021/acs.chemmater.8b03612.

[ref25] Yano H.; Watanabe M.; Iiyama A.; Uchida H. Particle-size effect of Pt cathode catalysts on durability in fuel cells. Nano Energy 2016, 29, 323–333. 10.1016/j.nanoen.2016.02.016.

[ref26] Di Noto V.; Negro E. Development of nano-electrocatalysts based on carbon nitride supports for the ORR processes in PEM fuel cells. Electrochim. Acta 2010, 55 (26), 7564–7574. 10.1016/j.electacta.2009.11.032.

[ref27] Di Noto V.; Negro E.; Polizzi S.; Vezzù K.; Toniolo L.; Cavinato G. Synthesis, studies and fuel cell performance of “core–shell” electrocatalysts for oxygen reduction reaction based on a PtNix carbon nitride “shell” and a pyrolyzed polyketone nanoball “core. international journal of hydrogen energy 2014, 39 (6), 2812–2827. 10.1016/j.ijhydene.2013.08.054.

[ref28] Di Noto V.; Negro E. Synthesis, characterization and electrochemical performance of tri-metal Pt-free carbon nitride electrocatalysts for the oxygen reduction reaction. Electrochimica acta 2010, 55 (4), 1407–1418. 10.1016/j.electacta.2009.06.009.

[ref29] Di Noto V.; Negro E.; Nale A.; Kulesza P. J.; Rutkowska I. A.; Vezzù K.; Pagot G. Correlation between Precursor Properties and Performance in the Oxygen Reduction Reaction of Pt and Co “Core-shell” Carbon Nitride-Based Electrocatalysts. Electrocatalysis 2020, 11 (2), 143–159. 10.1007/s12678-019-00569-8.

[ref30] Negro E.; Polizzi S.; Vezzu K.; Toniolo L.; Cavinato G.; Di Noto V. Interplay between morphology and electrochemical performance of “core–shell” electrocatalysts for oxygen reduction reaction based on a PtNix carbon nitride “shell” and a pyrolyzed polyketone nanoball “core. international journal of hydrogen energy 2014, 39 (6), 2828–2841. 10.1016/j.ijhydene.2013.08.053.

[ref31] Vezzù K.; Bach Delpeuch A.; Negro E.; Polizzi S.; Nawn G.; Bertasi F.; Pagot G.; Artyushkova K.; Atanassov P.; Di Noto V. Fe-carbon nitride “Core-shell” electrocatalysts for the oxygen reduction reaction. Electrochim. Acta 2016, 222, 1778–1791. 10.1016/j.electacta.2016.11.093.

[ref32] Zhang W.; Cheng R.-R.; Bi H.-H.; Lu Y.-H.; Ma L.-B.; He X.-J. A review of porous carbons produced by template methods for supercapacitor applications. New Carbon Materials 2021, 36 (1), 69–81. 10.1016/S1872-5805(21)60005-7.

[ref33] Di Noto V.; Negro E.; Gliubizzi R.; Gross S.; Maccato C.; Pace G. Pt and Ni Carbon Nitride Electrocatalysts for the Oxygen Reduction Reaction. J. Electrochem. Soc. 2007, 154 (8), B745 10.1149/1.2740019.

[ref34] Callejas-Tovar R.; Diaz C. A.; de la Hoz J. M. M.; Balbuena P. B. Dealloying of platinum-based alloy catalysts: Kinetic Monte Carlo simulations. Electrochim. Acta 2013, 101, 326–333. 10.1016/j.electacta.2013.01.053.

[ref35] Negro E.; Vezzù K.; Bertasi F.; Schiavuta P.; Toniolo L.; Polizzi S.; Di Noto V. Interplay between Nitrogen Concentration, Structure, Morphology, and Electrochemical Performance of PdCoNi ″Core-Shell″ Carbon Nitride Electrocatalysts for the Oxygen Reduction Reaction. ChemElectroChem. 2014, 1 (8), 1359–1369. 10.1002/celc.201402041.

[ref36] Di Noto V.; Pagot G.; Negro E.; Vezzù K.; Kulesza P. J.; Rutkowska I. A.; Pace G. A formalism to compare electrocatalysts for the oxygen reduction reaction by cyclic voltammetry with the thin-film rotating ring-disk electrode measurements. Current Opinion in Electrochemistry 2022, 31, 100839 10.1016/j.coelec.2021.100839.

